# Assessing the Efficacy of 18F FDG PET-CT in Preoperative Staging of Early-Stage Cervical Cancer in Novi Sad, Serbia—A Pilot Study

**DOI:** 10.3390/jcm13237445

**Published:** 2024-12-06

**Authors:** Goran Malenković, Jelena Malenković, Sanja Tomić, Armin Šljivo, Slobodan Tomić

**Affiliations:** 1 Faculty of Medicine, University of Novi Sad, 21000 Novi Sad, Serbia; goran.malenkovic@mf.uns.ac.rs (G.M.); jelenam@gmail.com (J.M.); sanja.tomic@mf.uns.ac.rs (S.T.); 2 ASA Institute for Research and Development and Innovation, 71 000 Sarajevo, Bosnia and Herzegovina; sljivo95@windowslive.com

**Keywords:** cervical carcinoma, diagnosis, staging, Serbia, 18F FDG PET-CT

## Abstract

**Background and Objectives**: This study primarily aims to evaluate the preoperative staging effectiveness of PET-CT in early-stage cervical cancer, particularly, its ability to detect primary tumors and micrometastases. **Materials and Methods**: In this retrospective study, cervical cancer patients who had undergone preoperative 18F FDG PET-CT scans and were treated at the Department of Gynecology, Institute of Oncology, Vojvodina, in Sremska Kamenica, during the period from 2016 to 2020 were analyzed. **Results**: The study included 62 patients (mean age, 49.3 ± 9.6 years). Squamous cell carcinoma was the predominant histological type (95.2%), with G2 differentiation (82.3%) and FIGO stage Ib1 (80.6%) being the most common. Assessed by 18F FDG PET-CT, the mean tumor size was 26.4 ± 10.8 mm, which is slightly lower than the 26.9 mm measured during clinical examination (*p* = 0.784), with a significant (r = 0.678, *p* < 0.001) correlation between these methods. 18F FDG PET-CT demonstrated an overall accuracy of 88.7% for identifying primary tumors, with a sensitivity of 86.8%, specificity of 100.0%, PPV of 100.0%, and NPV of 56.2%. An intraoperative examination showed identical overall accuracy but higher sensitivity (98.1%) and lower specificity (33.3%). For 18F FDG PET-CT, the level of agreement with the histopathological examination was good (Kappa 0.656), while for the intraoperative examination, it was moderate (Kappa 0.409). Regarding the lymph node assessment, 18F FDG PET-CT’s accuracy was 82.2%, with a sensitivity of 53.8% and a specificity of 89.8%. The intraoperative examination showed lower accuracy (66.1%) but higher sensitivity (76.9%). The 18F FDG PET-CT Kappa value indicated moderate agreement (0.449), while the intraoperative examination showed poor agreement (0.282). **Conclusions**: In conclusion, significant effectiveness is shown by 18F FDG PET-CT for preoperative staging of early-stage cervical cancer, offering superior accuracy in detecting primary tumors and micrometastases, particularly in predicting lymph node metastases, thereby enhancing diagnostic accuracy and informing treatment decisions.

## 1. Introduction

Cervical carcinoma remains a significant public health challenge worldwide, ranking as the third most common gynecologic malignancy, despite advances in early detection diagnostic techniques, enhancements in preventive programs, and an extended pre-invasive latency period. The estimated global incidence is 569,847 new cases per year [1]. According to 2018 epidemiological data from Serbia, there were 1327 new cases of cervical carcinoma diagnosed annually, making it the fourth most common cancer among women and the second most prevalent cancer in females aged 15 to 44 years [2]. Achieving accurate staging in cervical cancer has long been challenging, as previous staging systems like the FIGO 2009 classification excluded lymph node involvement and remote metastases [3]. The 2018 FIGO update introduced imaging techniques to address these gaps, enhancing staging accuracy [4,5]. While CT and MRI offer non-invasive diagnostic benefits, they primarily assess anatomical features. The updated FIGO classification emphasized ultrasound as essential for accurate cervical cancer staging, noting that two-dimensional and three-dimensional transvaginal ultrasounds effectively assess parametrial infiltration and offer imaging insights comparable to MRI, thus enhancing staging precision by providing a comprehensive view of cervical regions [6]. In contrast, 18F-FDG-PET-CT identifies hypermetabolic activity associated with malignancy, making it especially effective for identifying micrometastases in paraaortic and pelvic lymph nodes. This improved specificity allows for more precise staging and tailored therapeutic approaches, positioning 18F FDG PET-CT as an important asset in cervical cancer treatment [3].

The 18F FDG PET-CT imaging technique is crucial for detecting lymph node metastases in cervical cancer [3,4,5] and enables the precise identification of malignant tissues during radiation therapy, while also assisting in surgical planning to ensure complete tumor removal with minimal damage to adjacent healthy tissues. Furthermore, PET CT is pivotal in evaluating treatment responses following chemotherapy or radiation therapy [5]. Despite its recognized utility, the diagnostic accuracy of this technique in early-stage tumors suitable for resection remains inadequately studied; however, it is considered the preferred imaging method for examining the lymph node status in patients with suspected recurrence and stage II or greater disease. The effectiveness of this method in patients with early-stage, resectable tumors has not been thoroughly investigated; however, it is considered the gold standard for diagnosing nodal status in individuals with stage II and/or higher cervical cancer disease, as well as in those at an increased risk for recurrence [5]. Numerous studies have investigated the diagnostic utility of 18 FDG PET-CT, as well as CT and MRI imaging in the preliminary evaluation of cervical cancer staging and their influence on guiding the selection of primary therapeutic strategies [7,8,9,10].

Our study primarily aimed to assess the diagnostic effectiveness of 18F FDG PET-CT in the preoperative staging of early-stage cervical carcinoma, with a particular emphasis on its ability to accurately detect primary tumors and micrometastatic lesions. This investigation aims to define the function of this imaging technique in assessing disease extent, thereby guiding clinical decision making regarding surgical interventions and adjuvant therapies, ultimately improving the prognosis of cervical cancer patients.

## 2. Materials and Methods

This was a retrospective study, which included patients who received surgical treatments at the Department of Gynecology, Clinic for Operative Oncology at the Institute of Oncology, Vojvodina, in Sremska Kamenica, in the period of 2016–2020, with data obtained from their medical records. The inclusion criteria for the patient cohort were the following: individuals aged 18–65 years with pathohistological confirmed invasive cervical carcinoma, and classified as having a primarily operable disease, specifically within FIGO stages such as Ia2, Ib1, Ib2, IIa1, and early IIb. All participants received 18F FDG PET-CT imaging 7–14 days before the surgery for the preoperative evaluation of disease extent. The exclusion criteria were the following: individuals with a history of malignant diseases, those who had received therapy as part of a prior treatment protocol for cervical carcinoma before the surgical intervention, and those with chronic conditions contraindicating surgical treatment.

### 2.1. Methods

The evaluation and comparison of cervical carcinoma progression were conducted using preoperative clinical assessments alongside 18F FDG PET-CT imaging, followed by intraoperative evaluations and a histopathological analysis of the excised specimens.

### 2.2. Clinical Examination

According to clinical examination criteria, including examinations with speculum and bimanual assessments of the vagina and rectum, the primarily operable stages included Ia2, IIa, Ib1, Ib2, and early IIb, which are marked by the initial invasion of the lateral parametria.

### 2.3. 18 F-FDG-PET-CT

A Biograph 64 True Point system (Siemens Medical Systems^®^, Erlangen, Germany) was used to perform the 18F FDG PET-CT scan. Participants were given extensive guidelines for preparation and an intravenous injection of 18F FDG, with body-weight-based dosages, typically in the range of 185–370 MBq (approximately 4.07 MBq per kilogram). After a 60 to 90 min uptake period in a radiation-protected room where patients voided their bladders, the imaging commenced with a CT scout scan to establish the range, typically extending from the base of the skull to mid-thigh, across 6 to 7 positions on the PET acquisition table. This initial scan was succeeded by a comprehensive CT scan aimed at attenuattable ion correction and the morphological evaluation of lesions. After acquiring the PET data, the positron emission detector system reconstructed the CT and corrected PET tomoscintigrams in axial, sagittal, and coronal views, which were then fused using specialized software for analysis in all three orientations alongside the uncorrected PET tomoscintigrams. The status of lymph nodes in the paraaortic and pelvic regions, along with the detection of distant metastases, was carefully assessed. During reconstruction, attenuation and scatter corrections were applied to both the CT and PET images, which were then volumetrically reconstructed and presented in axial, sagittal, and coronal views to facilitate thorough analysis. Tumors were identified through PET-CT scans utilizing manually outlined regions of interest. Lymph node status assessment, essential for FIGO staging of cervical cancer via 18F FDG PET-CT, included enlarged lymph nodes suspected of malignancy, following relevant AJCC classification guidelines.

### 2.4. Intraoperative Examination

For patients with FIGO stage IA2 to IIB cervical cancer deemed eligible for surgery, a radical hysterectomy was performed via an abdominal incision using paraumbilical, lower and/or upper medial laparotomy techniques. A thorough examination of the pelvic and abdominal organs, including lymph nodes, assessed cervix size and cancer invasion into the parametria, rectum, and bladder. Palpable lymph nodes larger than 1 cm were subjected to histopathological analysis, while the surgical stage of the disease was determined based on intraoperative findings, tumor size, parametrial involvement, vaginal invasion, and lymph node status.

### 2.5. Pathohistological Examination

The pathologist conducted an examination of the surgically excised specimen, providing a report on various characteristics, including tumor size, type, histological differentiation, the depth of stromal invasion, infiltration of the parametria, lymphovascular invasion, involvement of the vagina, and the total and metastatic lymph node counts from various regions, while postoperative cervical cancer staging was performed according to AJCC classification using these results, which were also analyzed with clinical data, preoperative 18F FDG PET-CT findings, and intraoperative observations.

### 2.6. Statistical Analysis

Using SPSS (v. 26), the statistical analysis was performed with descriptive statistics for quantitative and qualitative variables, where normally distributed data were summarized with absolute and relative frequencies, arithmetic mean, and standard deviation, and non-normally distributed data were reported as median and interquartile range (IQR, 25–75%). The Kappa statistic and McNemar’s test were employed to evaluate the agreement between the diagnostic approaches, while a paired-samples *t*-test analyzed the differences between the methods. Pearson’s correlation assessed the relationships between the diagnostic methods, and sensitivity, specificity, the positive predictive value (PPV), the negative predictive value (NPV), and the overall accuracy were computed to evaluate the diagnostic efficacy of the tests.

## 3. Results

The sample involved 62 patients, with a mean age of 49.3 ± 9.6 years (range, 32–75 years). The predominant histological type of cervical carcinoma identified through the pathohistological analysis of biopsies, cervical canal curettage, and/or conization was squamous cell with 59 (95.2%), followed by adenocarcinoma with 2 (3.2%) and adenosarcoma with 1 (1.6%). The most frequently observed grade was G2 51 (82.3%), followed by G3 6 (9.7%), and G1 5 (8.1%). The most common clinical stage was Ib1 with 50 (80.6%), followed by Ib2 with 5 (8.1%). All other clinicopathological characteristics of the patients are presented in Table 1.

### 3.1. Comparative Analysis of Tumor Dimensions: Clinical Examination vs. 18F FDG PET-CT Imaging

**Figure 1:**
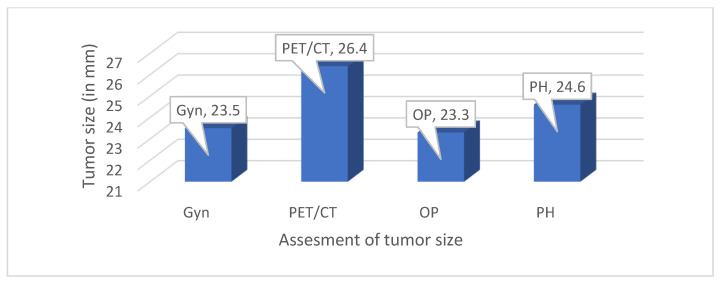
Mean tumor diameter—clinical examination, PET-CT scan, intraoperative examination, and pathological examination of surgical material.

In 46 patients, the mean tumor diameter measured by 18F FDG PET-CT was 26.4 ± 10.8 mm, but the tumor size could not be determined in 16 patients, and vaginal involvement was identified in only 2 patients (3.2%). In 43 patients, tumor dimensions assessed by clinical examination and 18F FDG PET-CT showed a lower mean diameter for the imaging technique compared to that for the clinical examination (26.4 mm vs. 26.9 mm; *p* = 0.784). Furthermore, a strong linear correlation was observed between the tumor dimensions derived from 18F-FDG-PET-CT and those measured during clinical examinations (r = 0.678, *p* < 0.001). The mean tumor sizes measured by various examinations are shown in Figure 1.

The overall prediction accuracy of 18F-FDG-PET-CT was 88.7% (55/62), which was identical to that of the intraoperative examination. However, 18F FDG PET-CT showed a sensitivity of 86.8% (46/53) (95% CI, 79.7–93.9%), while intraoperative examination demonstrated a higher sensitivity of 98.1% (52/53) (95% CI, 95.3–100.0%). In terms of specificity, 18F-FDG-PET-CT achieved 100% (9/9) (95% CI, 100–100%), whereas the intraoperative examination had a lower specificity of 33.3% (3/9) (95% CI, 23.5–43.2%). The PPV for 18F-FDG-PET-CT was 100% (46/46) (95% CI, 100–100%), compared to 89.7% (52/58) (95% CI, 83.3–96.0%) for the intraoperative examination. The NPV was 56.2% (9/16) (95% CI, 45.9–66.6%) for 18F-FDG-PET-CT and 75.0% (3/4) (95% CI, 66.0–84.0%) for the intraoperative examination. False negatives were more prevalent in 18F-FDG-PET-CT, occurring in 43.8% (7/16) (95% CI, 33.4–54.1%) of cases, while the intraoperative examination had a lower false negative rate of 25.0% (1/4) (95% CI, 16.0–34.0%). There were no false positives recorded for 18F-FDG-PET-CT, while the intraoperative examination had a false positive rate of 10.3% (6/58) (95% CI, 4.0–16.7%). Finally, the agreement with the histopathological examination was reflected by a Kappa value of 0.656 for 18F-FDG-PET-CT, indicating good agreement, compared to a Kappa value of 0.409 for the intraoperative examination, which indicated moderate agreement.

All correlations between the mean tumor size measurements obtained through clinical examinations, PET-CT scans, intraoperative examinations, and histopathological examinations of the surgical material were above 0.600 and were statistically significant. The strongest correlation of tumor size was observed between the clinical examination and intraoperative examination (r = 0.821), as well as between the PET-CT scan and intraoperative examination (r = 0.752). All differences between the mean tumor size measurements were statistically highly insignificant. The smallest difference in mean tumor size was between the PET-CT scan and intraoperative examination (*p* = 0.891) and between the PET-CT scan and histopathological examination of the surgical material (*p* = 0.799) (Table 2 and Table 3).

### 3.2. Comparative Evaluation of Lymph Node Metastases Using 18F-FDG-PET-CT, Intraoperative Findings, and Pathohistological Examination 

18F-FDG-PET-CT identified suspected metastatic activity in the right hemipelvis lymph nodes of six patients (9.7%), averaging 12.0 ± 6.54 mm (range: 8–25 mm). In the left hemipelvis, nine patients (14.5%) showed similar findings, with a mean size of 9.67 ± 1.80 mm (range: 8–12 mm). Additionally, 18F-FDG-PET-CT detected suspected metastases in the paraaortic lymph nodes of one patient, measuring 6 mm.

In 22 patients (35.5%), the intraoperative assessment indicated potential tumor involvement in lymph nodes across 32 locations (obturator—13 (7 + 3 + 3); iliaca communis—12 (4 + 3 + 5); iliaca externa—7 (4 + 3)), in the right hemipelvis. Among these, 18 patients had one suspicious lymph node/location and 4 patients had two (a mean of 1.82 lymph nodes per patient). In 22 patients (35.5%), the intraoperative assessment indicated potential tumor involvement in lymph nodes across 31 locations (obturator—10 (5 + 3 + 2); iliaca communis—12 (4 + 2 + 6); iliaca externa—9 (4 + 3 + 2), in the left hemipelvis. Of these, 18 patients had one suspicious lymph node per location, while four patients had two, resulting in a mean of 1.82 lymph nodes per patient. Intraoperative findings indicated suspected involvement of paraaortic lymph nodes in 2 patients (3.2%), while 17 patients showed potential lymph node involvement in both hemipelvises and 5 in either the right or left hemipelvis.

The presence of metastases in the lymph nodes of the right hemipelvis was confirmed by a pathohistological analysis in nine (14.5%) patients across 11 locations (obturator—3 (1 + 2); iliaca communis—6 (1 + 1 + 4); iliaca externa—2 (1 + 1)). Among these, six patients had one metastatic lymph node per location, one patient had two, and two had three (mean two metastatic lymph nodes/patient). In the left hemipelvis, eight patients (14.5%) had pathohistologically confirmed metastases across nine locations (obturator—four; iliaca communis—four (one + three); iliaca externa—one). Of these, six patients had one metastatic lymph node, one patient had two, and one patient had eight metastatic lymph nodes per location (a mean of two metastatic lymph nodes/patient). The pathohistological examination confirmed metastases in the paraaortic lymph nodes in one (1.6%) patient with seven metastatic nodes, in both hemipelvises in four patients, and in either the left or right hemipelvis in four and five patients, respectively. All other data regarding the comparison of suspected lymph node involvement by localization and examination type are presented in Table 4.

The overall accuracy of 18F-FDG-PET-CT in predicting lymph node involvement was 82.2% (51/62), higher than the 66.1% (41/62) for the intraoperative examination. Although 18F-FDG-PET-CT had a lower sensitivity of 53.8% (7/13) (95% CI, 43.4–64.2%) compared to the intraoperative examination’s 76.9% (10/13) (95% CI, 68.1–85.7%), its specificity was markedly higher at 89.8% (44/49) (95% CI, 83.5–96.1%) versus 63.3% (31/49) (95% CI, 53.2–73.3%). The PPV for 18F-FDG-PET-CT was 58.3% (7/12) (95% CI, 48.0–68.6%), while the intraoperative examination’s PPV was only 35.7% (10/28) (95% CI, 25.7–45.7%). The NPV was 88.8% (44/50) (95% CI, 81.2–94.8%) for 18F-FDG-PET-CT and 91.2% (31/34) (95% CI, 85.2–97.1%) for the intraoperative examination.

False negatives were slightly higher for 18F-FDG-PET-CT at 12.0% (6/50) (95% CI, 5.22–18.8%) compared to 8.82% (3/34) (95% CI, 2.90–14.8%) for the intraoperative examination. However, false positives were lower for 18F-FDG-PET-CT at 41.7% (5/12) (95% CI, 31.4–52.0%) versus a significant 64.3% (18/28) (95% CI, 54.3–74.3%) for the intraoperative examination. The Kappa value for 18F-FDG-PET-CT indicated moderate agreement (0.449), while the intraoperative examination reflected a poor agreement (0.282). The McNemar test results showed a *p* = 1.000 for 18F-FDG-PET-CT, suggesting symmetry in deviations from agreement, while the intraoperative examination had a *p*-value of 0.001, indicating asymmetry in its deviations.

## 4. Discussion

The study evaluated patients mainly diagnosed with squamous cell carcinoma, mostly with moderate histological differentiation and those classified at FIGO stage Ib1. Both 18F FDG PET-CT and intraoperative findings displayed similar overall prediction accuracies for assessing primary tumors. However, 18F FDG PET-CT had lower sensitivity but higher specificity compared to intraoperative examinations. Its positive predictive value was markedly superior, indicating strong effectiveness in confirming true positive cases, while the intraoperative examination provided a better negative predictive value. Notably, when predicting lymph node involvement, 18F FDG PET-CT outperformed the intraoperative examination in overall accuracy, despite having a higher false negative rate. In contrast, the intraoperative examination had a lower false positive rate. The level of agreement with histopathological findings was poor for the intraoperative examination, while moderate for 18F FDG/-CT.

The treatment for cervical cancer combines surgery and radiotherapy, where accurately assessing tumor expansion is critical for determining the most effective therapeutic approach. Research indicates that 18F FDG PET-CT has a significant position in this context, with the potential to alter the initial therapeutic plan in up to 30% of patients [9,10]. This highlights the significance of advanced imaging techniques in personalizing treatment strategies, which vary based on disease stage and encompass minimally invasive surgical interventions that preserve female fertility alongside chemoradiotherapy for locally advanced disease [9,11,12]. Metastasis status in para-aortic and pelvic lymph nodes is a critical prognostic factor in early-stage cervical cancer, significantly influencing patient outcomes. Research has consistently shown that patients with lymph node metastases exhibit markedly lower survival rates compared to those without metastases [9,11,12,13,14,15,16,17,18,19]. This relationship underscores the vital role of the accurate assessment of lymph node involvement in treatment planning and prognostication. The presence of metastases can indicate a more aggressive disease course and may necessitate more intensive treatment strategies, including chemoradiotherapy, in addition to surgical intervention. Consequently, the thorough evaluation of lymph node status using advanced imaging techniques and pathological examination is essential for optimizing individualized treatment approaches and improving survival outcomes for patients with early-stage cervical carcinoma.

This study found that 18F FDG PET-CT had an overall accuracy of 88.7% in predicting primary cervical cancer, followed by a sensitivity of 86.8% and specificity of 100%. The accuracy of 18F FDG PET-CT in predicting lymph node involvement was 82.2%, with a sensitivity of 53.8% and a specificity of 89.8%. These findings are supported by a meta-analysis showing that the sensitivity and specificity of 18F FDG PET-CT for detecting metastases in cervical cancer lymph nodes are 82% and 95%, respectively [20]. However, the discrepancies in sensitivity could stem from variations in patient populations; for instance, differences in disease stages, histological types, or the characteristics of lymph node involvement could influence the performance of the imaging modality. Additionally, the methodologies employed in both studies might differ, such as the criteria for defining lymph node metastases, the timing of imaging in relation to surgery, or variations in the interpretation of PET-CT results by radiologists. Moreover, the sample size in each study can also play a crucial role in determining the robustness of the findings. A smaller sample size, as might be the case in our study, can lead to greater variability in results and potentially lower sensitivity. Conversely, Choi et al.’s meta-analysis likely aggregated data from multiple studies, which may have enhanced the reliability of their sensitivity and specificity estimates.

In our study, we observed no false positive results, although we did encounter instances of false negatives. Specifically, 18F FDG PET-CT imaging failed to identify the primary tumor in seven cases, with tumor sizes ranging from 2 to 14 mm. This occurrence is explained by the lower spatial resolution of this imaging technique, which may result in false negative findings for tumors smaller than one centimeter, as well as for tumors that invade the surface. In contrast, micrometastatic deposits are more readily identified using MRI [19]. The high false negative result rate may further be explained by the significant number of positive lymph nodes identified through histopathological examination that were smaller than 10 mm in diameter, alongside the presence of micrometastases, a small cluster of cancer cells that remains undetectable by both CT and MRI. Additionally, lymph nodes that show reactive changes due to hyperplasia or infection may contribute to false positive findings [20]. The standard criterion for identifying lymphadenopathy is a short-axis diameter larger than 10 mm. However, studies have indicated that over 80% of metastatic lymph nodes measure less than 10 mm, and more than 50% are smaller than 5 mm [11]. Sensitivity may be enhanced by adopting a threshold of 5 mm; however, the detection of small lymph nodes can also result in numerous false positive cases, thereby decreasing specificity [21].

The detection of micrometastases is paramount in determining treatment strategies for cervical cancer, as the presence of lymph node involvement can indicate a more aggressive disease course. This underscores the necessity for intensified treatment approaches that may include a combination of surgery, chemotherapy, and radiotherapy, depending on the extent of the metastasis. If lymph nodes are found to be involved with cancer cells, it often alters the therapeutic plan, leading clinicians to consider more aggressive treatment regimens that aim to address the systemic nature of the disease, thereby improving the chances of long-term survival. Detecting micrometastases in the pelvic and paraaortic lymph nodes is essential in cervical cancer management, as nodal involvement critically influences disease staging, prognosis, and therapeutic decision making. Identifying micrometastatic spreads enables oncologists to refine treatment plans by considering intensified or extended radiation fields, or adjunctive systemic therapies, in patients with confirmed nodal metastasis [22]. This precision helps to avoid both undertreatment—potentially risking disease recurrence—and overtreatment, which may expose patients without nodal involvement to unnecessary toxicity. Moreover, PET-CT’s capacity for detecting even small-volume metastases is advantageous in preoperative planning. When micrometastases are identified, it may inform the decision to pursue an extended lymphadenectomy, thus ensuring a comprehensive resection of the involved lymphatic tissue. In this way, PET-CT contributes to a more individualized and optimized treatment approach, improving patient outcomes and aligning with modern oncologic strategies that prioritize both survival and life quality [23].

To ensure the best possible outcomes, a thorough evaluation of lymph node status is critical. This evaluation involves the use of advanced imaging techniques, such as 18F-FDG-PET-CT, in conjunction with traditional pathological examination [22,23]. 

In early-stage cervical cancer, where the disease may be confined to the cervix and surrounding tissues, the ability to detect micrometastases can significantly impact patient management. If metastases are identified early, it allows for timely interventions that can potentially alter the course of the disease. For example, patients with detected micrometastases may require more comprehensive treatment strategies that could include extended lymphadenectomy or adjuvant therapies to mitigate the risk of recurrence [24]. 

Furthermore, the role of 18F-FDG-PET-CT in enhancing the detection of metastases is especially crucial in the context of personalized medicine. In Novi Sad, Serbia, the integration of this advanced imaging modality into clinical practice emphasizes its potential to revolutionize therapeutic strategies for cervical cancer patients. By facilitating an earlier and more accurate detection of metastatic involvement, 18F-FDG-PET-CT enables clinicians to make informed decisions regarding treatment, thereby improving the overall management of the disease.

A key limitation of our study was its small sample size, which reduced the statistical power to detect subtle effects and limited the generalizability of the findings. With a larger cohort, we might have observed more nuanced patterns in lymph node involvement and treatment outcomes that could further refine the applications of 18F FDG PET-CT. Future studies with larger and more diverse patient populations are warranted to validate these results and strengthen the evidence base, ultimately guiding oncologists in optimizing treatment plans for cervical cancer patients.

The potential of 18F FDG PET-CT to transform therapeutic strategies cannot be overstated. It empowers oncologists to identify patients who might benefit from escalated treatment regimens while also allowing for the avoidance of overtreatment in patients without involvement of the lymph nodes. As the landscape of cervical cancer management evolves, the continued use of advanced imaging techniques like 18F FDG PET-CT will have an essential place in ensuring that patients receive the most effective and personalized care possible, ultimately improving survival and life quality for those affected by this challenging disease. Additionally, recent advancements in machine learning are poised to significantly enhance diagnostic precision in oncology. Radiomic analysis, as demonstrated in the 2020 AROMA pilot study, reliably identifies patients with ovarian cancer, and subsequent studies have confirmed its ability to predict responses to neoadjuvant chemotherapy in advanced cervical cancer. This emerging approach promises to support more personalized and effective treatment strategies, shaping the future of cancer care [25,26,27].

## 5. Conclusions

In conclusion, this study highlights the crucial role of 18F FDG PET-CT in cervical cancer management, especially for squamous cell carcinoma patients at FIGO stage Ib1. While 18F FDG PET-CT and intraoperative examinations have comparable accuracy in predicting primary tumors, the former offers superior specificity and PPVs, making it effective for confirming true positives. Its ability to detect lymph node involvement is critical, as metastasis significantly influences patient outcomes. The early identification of micrometastases via 18F-FDG-PET-CT enables tailored treatment strategies, enhancing survival rates. In Novi Sad, Serbia, the integration of this advanced imaging modality provides valuable insights for potentially enhancing therapeutic approaches, allowing for timely and informed decisions in patient care. As cervical cancer management continues to evolve, 18F-FDG-PET-CT will remain essential for delivering effective, personalized interventions, ultimately improving patient outcomes.

## Figures and Tables

**Table 1:** Cervical cancer patient profile: histological types and FIGO staging.

Histological Cancer Type	N (62)	%
Squamous cell	59	95.2
Adenocarcinoma	2	3.2
Adenosarcoma	1	1.6
Degree of histological differentiation		
G1	5	8.1
G2	51	82.3
G3	6	9.7
FIGO stage distribution		
Ia2	2	3.2
Ib1	50	80.6
Ib2	5	8.1
IIa1	1	1.6
IIb	4	6.5

**Table 2:** Correlations of tumor dimensions obtained through clinical examination, PET-CT scan, intraoperative examination, and pathological examination.

Tumor Size	Clinical Examination (23.5 ± 13.2 mm)	PET-CT Exam (26.4 ± 10.8 mm)	Intraoperative Examination (23.3 ± 12.9 mm)
PET-CT exam (26.4 ± 10.8 mm)	r = 0.678	-	-
Intraoperative examination (23.3 ± 12.9 mm)	r = 0.821	r = 0.752	-
Pathohistological examination (24.6 ± 13.2 mm)	r = 0.617	r = 0.619	r = 0.708

**Table 3:** Differences with paired t-tests of tumor dimensions obtained through clinical examination, PET-CT scan, intraoperative examination, and pathological examination.

Tumor Size	PET-CT Exam (26.4 ± 10.8 mm)	Intraoperative Examination (23.3 ± 12.9 mm)	Pathohistological Examination (24.6 ± 13.2 mm)
Clinical examination (23.5 ± 13.2 mm)	*p* = 0.784	*p* = 0.690	*p* = 0.440
PET-CT exam (26.4 ± 10.8 mm)	-	*p* = 0.891	*p* = 0.799
Intraoperative examination (23.3 ± 12.9 mm)	-	-	*p* = 0.668

**Table 4:** Comparison of suspected lymph node involvement by localization and examination type.

Examination Type		Region	Localization	
18F-FDG-PET-CT			Right hemipelvis	Left hemipelvis
	Lymph nodes (N,%)	Iliaca communis	5 (8.1)	6 (9.7)
		Obturator	1 (1.6)	3 (4.8)
		Other	6 (9.7)	9 (14.5)
	Size of suspected lymph nodes (M ± SD)		12 ± 6.5	9.7 ± 1.8
Intraoperative exam	Lymph nodes (N,%)	Iliaca communis-iliaca externa	4 (18.2)	4 (18.2)
		Iliaca communis-obturator	3 (13.6)	2 (9.1)
		Iliaca communis	5 (22.7)	6 (27.3)
		Iliaca externa-obturator	3 (13.6)	3 (13.6)
		Obturator	7 (31.8)	2 (9.1)
Pathohistological exam	Lymph nodes (N,%)	Iliaca communis-iliaca externa	1 (11.1)	1 (12.5)
		Iliaca communis-obturator	1 (11.1)	0 (0.0)
		Iliaca communis	4 (44.4)	3 (37.5)
		Iliaca externa-obturator	1 (11.1)	0 (0.0)
		Obturator	2 (22.2)	4 (50.0)

## Data Availability

Data are available upon request.
